# Unravelling the multi-scale structure–property relationship of laser powder bed fusion processed and heat-treated AlSi10Mg

**DOI:** 10.1038/s41598-021-85047-2

**Published:** 2021-03-19

**Authors:** P. Van Cauwenbergh, V. Samaee, L. Thijs, J. Nejezchlebová, P. Sedlák, A. Iveković, D. Schryvers, B. Van Hooreweder, K. Vanmeensel

**Affiliations:** 1 3D Systems Leuven, Grauwmeer 14, 3001 Leuven, Belgium; 2 Department of Physics, Electron Microscopy for Materials Science (EMAT), University of Antwerp, Groenenborgerlaan 171, 2020 Antwerp, Belgium; 3 Institute of Thermomechanics, Academy of Sciences of the Czech Republic, Dolejškova 5, 182000 Prague, Czech Republic; 4 Department for Nanostructures Materials, Jozef Stefan Institute, Jamova c. 39, 1000 Ljubljana, Slovenia; 5 Department of Mechanical Engineering (MaPS), KU Leuven, Celestijnenlaan 300, 3001 Leuven, Belgium; 6 Department of Materials Engineering, KU Leuven, Kasteelpark Arenberg 44, 3001 Leuven, Belgium

**Keywords:** Metals and alloys, Aerospace engineering

## Abstract

Tailoring heat treatments for Laser Powder Bed Fusion (LPBF) processed materials is critical to ensure superior and repeatable material properties for high-end applications. This tailoring requires in-depth understanding of the LPBF-processed material. Therefore, the current study aims at unravelling the threefold interrelationship between the process (LPBF and heat treatment), the microstructure at different scales (macro-, meso-, micro-, and nano-scale), and the macroscopic material properties of AlSi10Mg. A similar solidification trajectory applies at different length scales when comparing the solidification of AlSi10Mg, ranging from mould-casting to rapid solidification (LPBF). The similarity in solidification trajectories triggers the reason why the Brody-Flemings cellular microsegregation solidification model could predict the cellular morphology of the LPBF as-printed microstructure. Where rapid solidification occurs at a much finer scale, the LPBF microstructure exhibits a significant grain refinement and a high degree of silicon (Si) supersaturation. This study has identified the grain refinement and Si supersaturation as critical assets of the as-printed microstructure, playing a vital role in achieving superior mechanical and thermal properties during heat treatment. Next, an electrical conductivity model could accurately predict the Si solute concentration in LPBF-processed and heat-treated AlSi10Mg and allows understanding the microstructural evolution during heat treatment. The LPBF-processed and heat-treated AlSi10Mg conditions (as-built (AB), direct-aged (DA), stress-relieved (SR), preheated (PH)) show an interesting range of superior mechanical properties (tensile strength: 300–450 MPa, elongation: 4–13%) compared to the mould-cast T6 reference condition.

## Introduction

Laser Powder Bed Fusion (LPBF) is an Additive Manufacturing (AM) process that uses Computer-Aided Design (CAD) data to build an object layer-by-layer and is also known as Direct Metal Printing (DMP). For the past decade, prominent progress regarding LPBF processing has been established regarding process scalability, reliability, productivity, and part quality^1–4^. Thanks to these achievements, Aerospace and Automotive adopted the LPBF technology with a specific interest in high strength-to-weight alloys such as AlSi10Mg^5^. Although several authors^6–11^ have contributed to revealing the process-structure–property interrelationship for LPBF-processed and heat-treated AlSi10Mg, it is still far from fully understood. More specifically, an in-depth understanding of this threefold interrelationship at multiple length scales remains among the prominent scientific challenges in metal AM^12^. Namely, the lack of understanding of this interrelationship originates from the unique LPBF solidification conditions leading to an ultrafine and often metastable microstructure with a particular thermal history induced by the intense cyclic heating- and cooling patterns^1,5,13^. The lack of understanding of the process-microstructure-property relationship is likewise reflected in thermal post-processing of LPBF parts. Heat treatments for LPBF-processed metals are too often blindly adopted from conventional processing, for example, adopting a T6 heat treatment for LPBF-processed AlSi10Mg. Considering the fundamental difference in microstructure obtained after LPBF processing compared to conventional processing, one cannot expect these microstructures to evolve similarly during heat treatment. Therefore, these conventionally-adopted heat treatments are often suboptimal for LPBF-processed alloys and can result in inferior mechanical properties compared to conventionally processed parts. Consequently, the need for tailored heat treatments for LPBF-processed metals contributes to the maturity challenges of metal AM^12^.

Hence, this work aims to examine the threefold interrelationship between the process (LPBF and heat treatment), the multi-scaled microstructure, and the macroscopic material property response of LPBF-processed AlSi10Mg. This study discusses the solidification mechanisms establishing the morphological distinction within the meltpool (meso-scale). It applies the Brody-Flemings cellular microsegregation model based on local chemical distribution measurements to predict the cellular features in the meltpool centre (micro-scale). Moreover, this study compares the solidification trajectory followed during conventional mould-casting and rapid solidification. The paper explains the correlation between the macroscopic material property response and the multi-scale (meso-, micro-, and nano-scale) structural features and their evolution during heat treatment. The ability to predict the degree of Si supersaturation in LPBF-processed AlSi10Mg via an electrical conductivity model is verified with TEM EDX analysis. Finally, this work evaluates the extent of elastic and plastic anisotropic behaviour of LPBF-processed AlSi10Mg.

## Materials and experiments

### LPBF processing and heat treatment

In the present work, a 3D Systems DMP Flex 350 was used to build the samples. The DMP Flex 350 LPBF machine is equipped with a fiber laser (500 W) with Gaussian energy distribution. The samples were built in 60 µm layer thickness with 3D Systems' commercial process parameter set LaserForm AlSi10Mg(A)_Sv8, and with a volumetric energy density (VED) of 38.7 J/mm^3^. The same process parameter set was applied to build the preheated samples at 200 °C with a baseplate preheating prototype. LaserForm AlSi10Mg0.3 (A) powder supplied by 3D Systems with a powder particle size ranging from 10 to 63 µm was used to print the samples. The chemical compositions of both the AlSi10Mg powder and the printed part complied with the standards AMS 7018 and ASTM F3318, respectively. The relative density of the LPBF as-built AlSi10Mg was verified by analysing optical images at magnification 5× via the pixel count method with the ImageJ software. The average pixel density was 99.8% based on ten samples printed across the build plate. This study evaluates the AlSi10Mg material properties and microstructure for various conditions, displayed in Table 1. Besides the as-built (AB) condition, two heat treatments, direct ageing (DA) and stress relief (SR), were tailored for LPBF-processed AlSi10Mg and selected based on the author's previous work^11^. Next, LPBF processing with baseplate preheating at 200 °C (PH) was also included in the study. Finally, an as-cast T6 (AC T6) condition served as the reference for this study.

**Table 1:** Labels used for the respective sample conditions with details on the heat treatment procedure.

Label	Condition	Heat treatment procedure
AB	LPBF as-built	NA
DA	LPBF + direct ageing	170 °C, 6 h, Furnace cooling (FC)
SR	LPBF + stress relief	270 °C, 2 h, FC
PH	LPBF with baseplate preheating at 200 °C	NA
AC T6	As-cast + T6	540 °C, 8 h, Water quenching (WQ) 160 °C, 6 h, FC

### Computational modelling

#### Thermodynamic modelling

The equilibrium and Scheil solidification trajectory of AlSi10Mg0.3 and the solute concentration profiles (Si, Mg) were calculated with the Thermo-Calc 2020b software, using the TCAL5.1 database for Al alloys.

#### Thermal modelling

A numerical transient heat transfer analysis was carried out using COMSOL Multiphysics 5.2a software. This thermal model simulates a single-track bead of the LPBF processing of AlSi10Mg. A volumetric heat source with a Gaussian distribution was used to simulate the laser beam. The numerical model considers conduction and radiation as heat transfer mechanism and excludes fluid dynamics and phase transformations. The results of the numerical model were verified and calibrated through comparison to the experimental meltpool dimensions obtained with single-track experiments using the identical LPBF process parameter set. A detailed description of the used thermal model was already published by Iveković et al.^14^. The calibrated thermal model was applied to predict the temperature distribution, thermal gradients and cooling rates within the meltpool.

### Mechanical testing

Five tensile coupons per sample condition were built to conduct tensile testing. The respective vertical (Z) and horizontal (XY) coupons were printed and post-machined to round tensile coupons according to ASTM E8M specimen 4. An Instron 5985 tensile testing machine equipped with a load cell that can apply a maximum force of 250 kN was used to conduct the tensile tests. A strain rate of 0.015 mm/mm/min was applied until the yield point, after which a strain rate of 0.4 mm/mm/min was applied. Tensile testing was performed in compliance with ASTM E8/E8M. Elastic constants of the LPBF-processed samples at room temperature were measured by resonant ultrasound spectroscopy (RUS) using a contactless laser-based setup. The samples were placed in a temperature-regulated chamber filled with a low-pressure nitrogen atmosphere. An infrared pulsed Nd:YAG laser (Quantel ULTRA, nominal wavelength 1.064 µm, pulse duration 8 ns) generated the sample vibrations. On the opposite side of the sample, the scanning laser vibrometer (Polytec OFV 505) recorded the vibrations and enabled the scanning of the surface in a regular mesh. Sedlák et al. describe the experimental setup and the resonant spectra evaluation more in detail^15^. The resonant spectra were measured in a frequency range from 0.2 to 2.5 MHz.

### Thermo-physical characterisation

Thermo-physical properties of the samples were characterised based on Differential Scanning Calorimetry (DSC) and Electrical Resistivity (ER). An LPBF AB AlSi10Mg sample (500 mg) was used for DSC analysis and was performed on a TA Instruments DSC Q2000. A thermal cycle between 20  and 550 °C with a heating and cooling rate of 20 °C/min was applied in an argon atmosphere. Two consecutive thermal cycles were run for the DSC analysis. ER measurements were performed at room temperature using a four-point contact technique on a Burster Resistomat type 2302.

### Microscopy

Vertical cross-sections along the building direction were taken from the bulk material for the investigation of the microstructure at a multi-scale level, using the following microscopes: light optical microscope (LOM) (Nikon Eclipse MA 100), scanning electron microscopes (SEM) (XL30 FEG, Philips and Nanosem 450, FEI), and scanning transmission electron microscope (STEM) (Tecnai Osiris, FEI, Hillsboro, OR USA), the latter equipped with an X-FEG operating at 200 kV. Energy-dispersive X-ray Spectroscopy (EDX)—Annular Dark Field (ADF)-STEM was used for chemical mapping. The samples were mounted in resin and ground with silicon carbide paper with grit sizes from 320 to 4000. After grinding, the coupons were polished sequentially with 3 µm and 1 µm diamond suspension and etched during 12 s with 0.5 vol% HF. A FIB-SEM dual beam FEI Helios NanoLab 650 instrument was used for TEM sample preparations. Protected by an ion beam-assisted Pt protective layer, TEM lamellas were lifted out, mounted on TEM grids, and milled with a Ga^+^ ion beam of 30 kV/0.79 nA and lower currents, finishing with a final polishing step at 2 kV/39 pA to reach a thickness of about 100 nm.

## Results

### Macroscopic material properties

#### Elastic tensor

The Reuss mean elastic constants and moduli of AB, DA, and SR were determined via the RUS method. The AB sample exhibits a mean Young's modulus (E) of 67.7 GPa and a shear modulus (G) of 25.3 GPa. After DA, the elastic moduli increases (E = 70.2 GPa, G = 26.3 GPa). Finally after SR, the elastic moduli (E = 71.1 GPa, G = 26.5 GPa) are comparable to DA. The full elastic tensor, the mean Poisson ratio, and the anisotropy factor of the respective conditions are covered in the appendix Table A1. The AB, DA, and SR conditions reveal almost isotropic elastic behaviour (i.e. ${C}_{11}\approx {C}_{33}$, ${C}_{12} \approx {C}_{13}$, ${C}_{44} \approx \frac{C11-C12 }{2} \left[ \text{GPa} \right]$). Namely, the anisotropy factor is close to unity (1.04) for all conditions (AB, DA, SR). The anisotropy factor is defined as $A={\left(\frac{{v}_{max}^{qT}}{{v}_{min}^{qT}}\right)}^{2} [-]$, where ${v}_{max}^{qT} [\frac{m}{s}]$ and ${v}_{min}^{qT} [\frac{m}{s}]$ are the maximum and minimum quasi-transverse acoustic wave velocities, respectively. This definition of the anisotropy factor is valid for arbitrary material symmetry^16^. Therefore, the mean isotropic elastic constants describe well the materials elastic behaviour. Moreover, the small standard errors of the elastic constants reveal that the directional variations of those elastic constants are negligible.

#### Quasi-static tensile properties

**Figure 1:**
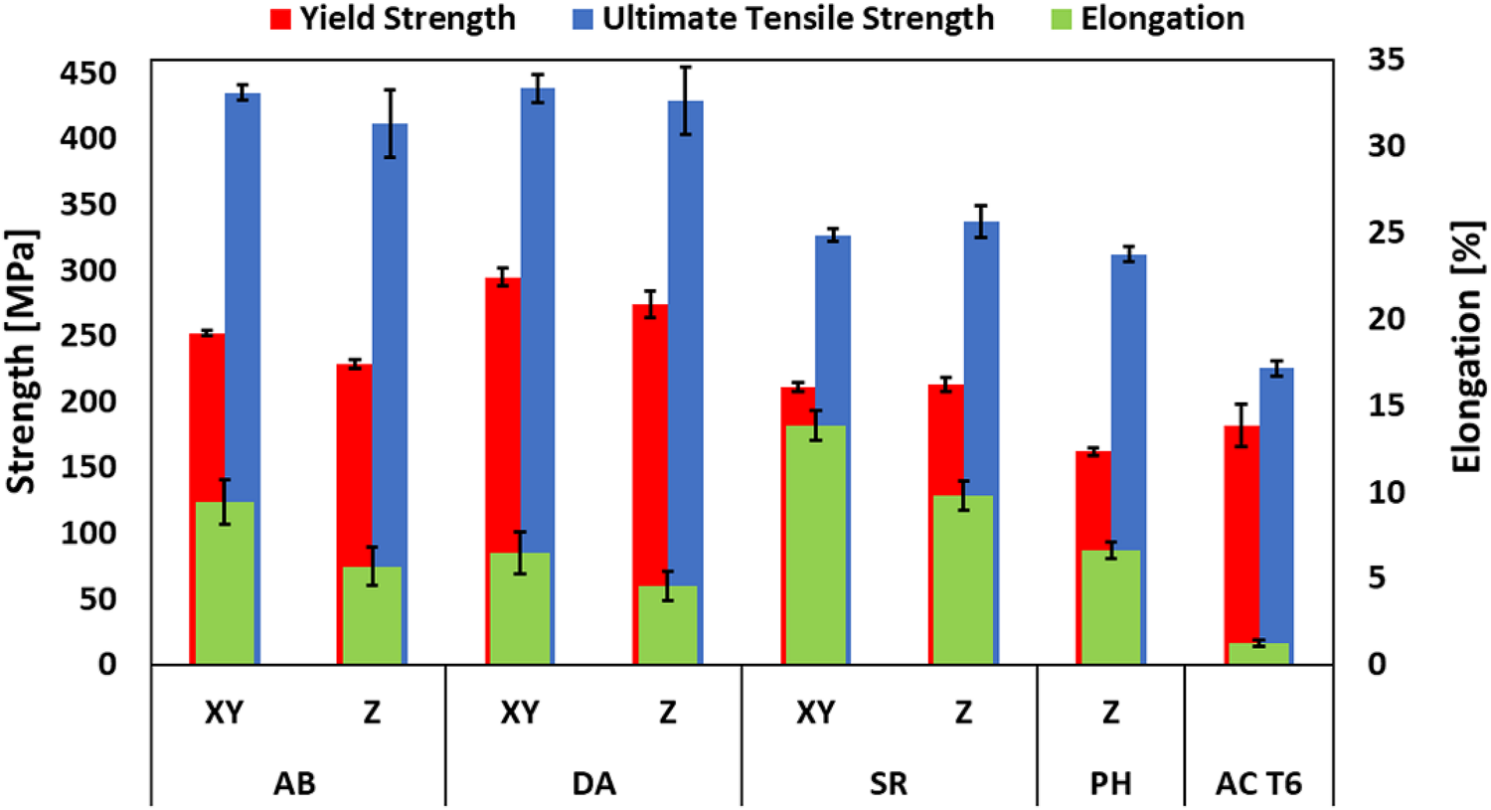
Tensile properties of LPBF-processed AlSi10Mg with conditions AB, DA, SR, PH, and their respective building orientations (horizontal (XY), vertical (Z)). Tensile properties of reference condition AlSi10Mg As-Cast T6 (AC T6). The average and 2σ standard error values are calculated from five tensile strength data points per condition.

**Figure 2:**
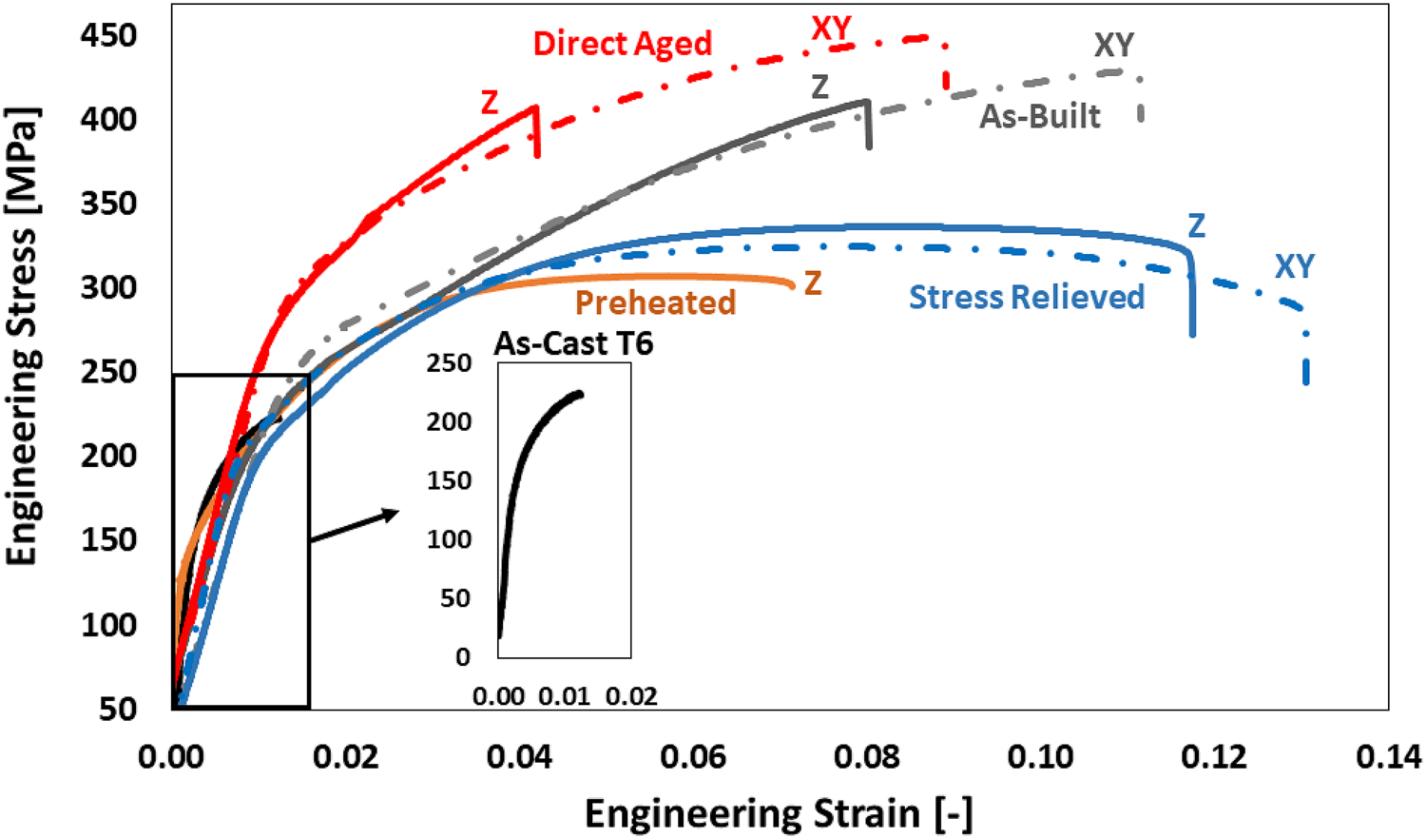
Engineering stress–strain curves of AlSi10Mg. LPBF-processed AlSi10Mg (AB, DA, SR, and PH) in horizontal (XY) and vertical (Z) building orientation; as-cast T6 (AC T6). For readability purposes, only one representative stress–strain curve is displayed per condition.

Figure 1 shows the average tensile properties based on five coupons, tested for each of the sample conditions (AB, DA, SR, PH, AC T6) and their orientations (XY, Z). Figure 2 displays the stress–strain curves of the respective conditions. For readability purposes, only one representative stress–strain curve is displayed per sample condition. The AB condition exhibits a yield strength (YS) of 250 MPa and a high ultimate tensile strength (UTS) up to 435 MPa. After DA, the YS increases to 295 MPa, whereas the UTS remains constant (440 MPa) compared to the AB samples. The SR condition shows a significant decrease in strength, YS (210 MPa) and UTS (335 MPa). The strength is even further reduced for the PH sample, attaining a YS of 160 MPa and UTS of 310 MPa. The AC T6 reference condition exhibits 180 MPa YS and 225 MPa UTS. Significant differences in plastic elongation between vertically and horizontally built samples are observed. For the AB, DA, and SR conditions, the elongation in the vertically built samples is notably lower than the horizontally built samples. Nonetheless, it is important to note that all LPBF conditions (AB, DA, SR, PH) show superior mechanical properties compared to the AlSi10Mg as-cast T6 reference condition (AC T6).

#### Thermo-physical properties

**Figure 3:**
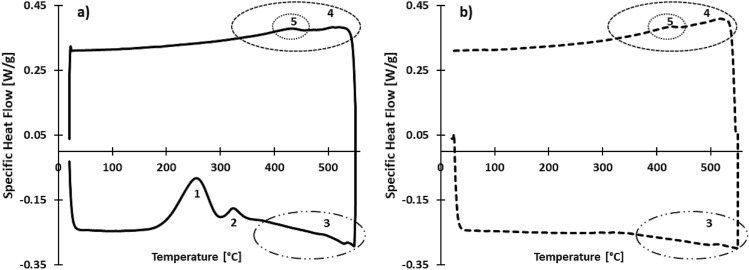
DSC cycles (heating and cooling rate: 20 °C/min) showing the specific heat flow curves of as-built AlSi10Mg: (**a**) first DSC cycle, (**b**) second DSC cycle.

A DSC measurement was conducted on an as-built AlSi10Mg sample, which underwent two consecutive thermal cycles (20–550 °C) with a heating and cooling rate of 20 °C/min. Figure 3a,b display the specific heat flow curves of the respective first and the second thermal cycles. A positive heat flow rate indicates an exothermic reaction. It is important to note that the DSC reaction peak temperature range depends on the DSC analysis's applied heating rate. Therefore, careful interpretation is required. The first DSC cycle shows four exothermic reaction peaks (1), (2), (4), (5) and one endothermic reaction peak (3). The reaction exothermic peaks (1) and (2) disappear in the second DSC cycle. The first two distinct exothermic peaks appear between 195 and 290 °C (1) and 305–350 °C (2). Next, the endothermic peak stretches from 350–550 °C (3). During cooling, two superimposed exothermic reaction peaks (4) and (5) can be noticed. The exothermic peak (4) ranges from 550 °C until 350 °C. Exothermic peak (5) ranges from 450 to 400 °C and is superimposed on the previous exothermic peak (4).

Table 2 summarises the electrical conductivity, *σ * [S/m], and thermal conductivity, *κ * [W/m K] of LPBF AlSi10Mg (AB, DA, SR, PH), measured at room temperature. The thermal conductivity values were calculated from the electrical conductivity values and rely on the Wiedemann–Franz law^17,18^. Thermal post-processing of the LPBF-processed AlSi10Mg enhances the thermal conductivity significantly from 124 W/m K, in as-built condition, up to 168 W/m K after SR.

**Table 2:** Electrical conductivity (σ) and thermal conductivity (κ) of LPBF AlSi10Mg (AB, DA, SR, PH), measured at room temperature.

	AB	DA	SR	PH
σ [10^6^ S/m]	17.4 ± 0.3	21.4 ± 0.7	23.5 ± 0.6	25.6 ± 0.4
κ [W/m K]	124 ± 2	153 ± 5	168 ± 4	183 ± 3

### Multi-scale microstructure

#### Mesostructure

**Figure 4:**
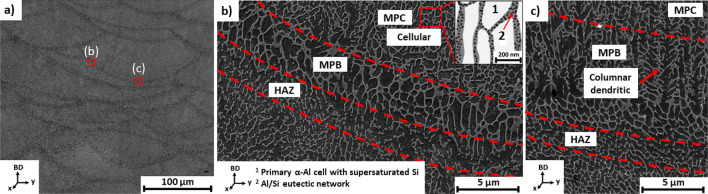
SEM images showing (**a**) the AB mesostructure presenting the overlapping meltpools in conduction mode; (**b**) and (**c**) magnified SEM images at the meltpool boundary showing the morphological transition of the microstructure between the meltpool centre (MPC—cellular), meltpool boundary (MPB—columnar dendritic), and heat-affected zone (HAZ).

**Figure 5:**
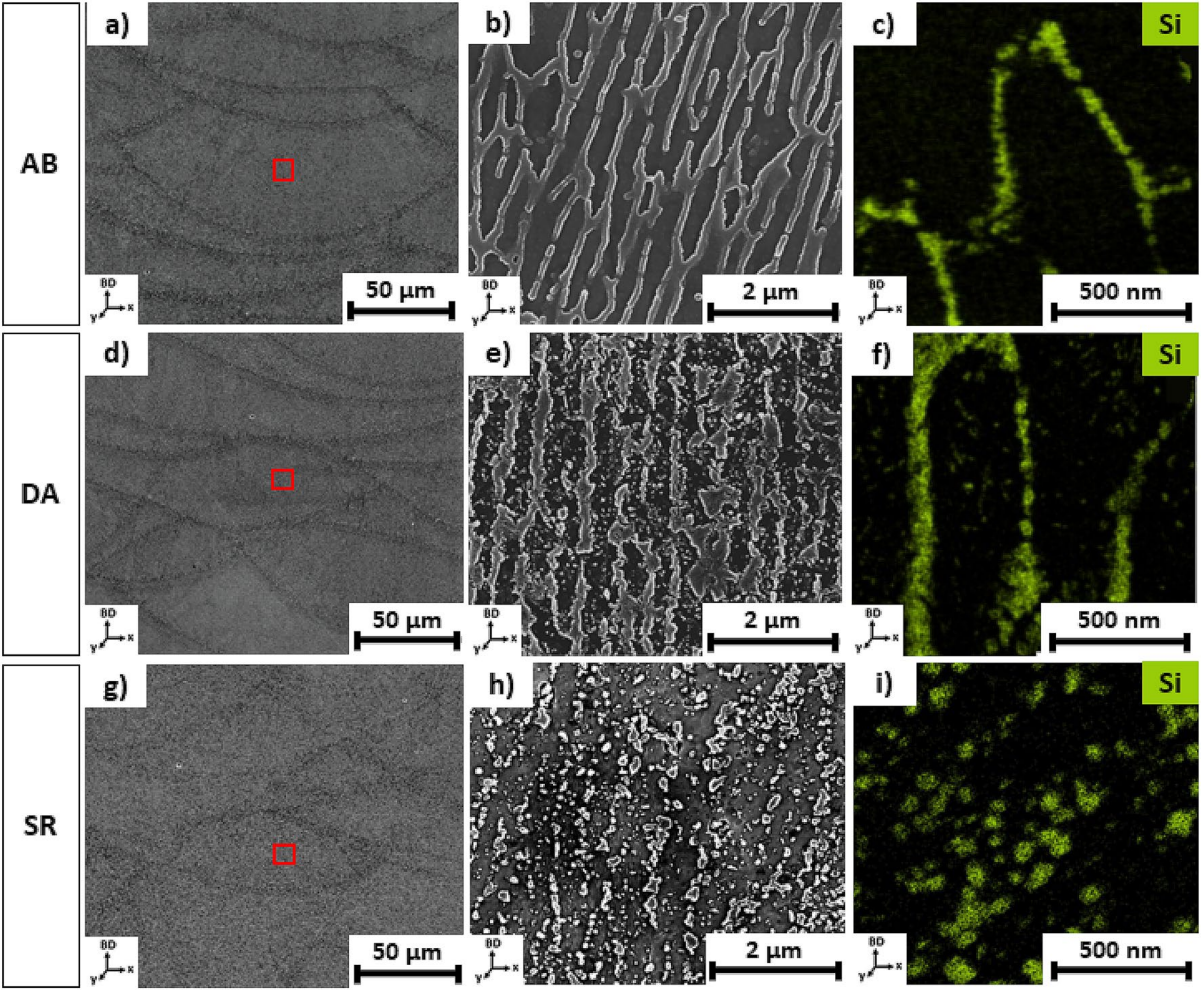
SEM images and Si STEM-EDX maps taken from the bulk material with vertical cross-sections along the building direction (BD) for the respective conditions: (**a**)–(**c**) as-built (AB); (**d**)–(**f**) direct-aged (DA); (**g**)–(**i**) stress-relieved (SR). The low-magnification images (**a**), (**d**), and (**g**) display the bulk mesostructure across several layers. The high-magnification images (**b**), (**e**), (**h**); and the Si STEM-EDX maps (**c**), (**f**), (**i**) are taken from the meltpool centre, indicated by the red-squared area.

Figure 4a displays the LPBF as-printed mesostructure originating from the bulk of the material, presenting overlapping meltpools in conduction mode. The dimensions of the meso- and micro-scaled structural features were manually quantified using the ImageJ software and a scale calibration based on the pixel aspect ratio of the image. The meltpools have an average meltpool width of 189 ± 15 µm and meltpool depth of 114 ± 8 µm. The meso-scale meltpools are visible because of the distinct morphological transition of the microstructure between the meltpool centre (MPC), meltpool boundary (MPB), and the heat-affected-zone (HAZ), as noticeable in Fig. 4b,c. The MPC's microstructure consists of a primary α-Al matrix exhibiting an ultrafine cellular morphology with an average cell size of 0.46 ± 0.09 µm and a Si-rich eutectic network in the intercellular region, as a secondary phase. The MPB exhibits a coarser microstructure of α-Al cells (0.89 ± 0.24 µm) mixed with columnar dendrites and with the Si-rich eutectic network surrounding these cells and dendrites. The HAZ shows a partial disintegration and spheroidisation of the Si eutectic network. Figure 5a,d,g show the evolution of the mesostructure for the respective conditions (AB, DA, and SR). AB and DA show no significant difference for the mesostructure. On the other hand, the mesostructure homogenises partially after SR, yet the meltpool boundaries are still noticeable in certain regions.

#### Microstructure

The microstructural evolution of the AB, DA, and SR conditions was evaluated based on the microstructural images and Si STEM-EDX mappings, taken from the bulk of the material and displayed in Fig. 5. The AB microstructure shows sub-micrometre-sized primary α-Al cells surrounded by the Al/Si eutectic network in the intercellular region, shown in Fig. 5a–c. After DA, ultrafine needle-like and cubic-like Si precipitates form in the primary Al cell while the eutectic network remains intact, as indicated in Fig. 5d–f. After SR, the microstructure evolves through the disintegration of the Al/Si eutectic network, followed by spheroidisation, thus obtaining coarse Si particles along the Al cell boundaries. Likewise, Si particles precipitate from the primary Al cell. The SR microstructure exhibits coarser Si particles with a less dense particle distribution when compared to DA. Thus, the SR microstructure results in a bi-modal Si particle distribution, exhibited in Fig. 5g–i. Besides the presence of a few Mg-rich Si co-precipitates, no evidence of the stable Mg_2_Si phase was found in any investigated microstructures.

#### Nanostructure

The extent of Si supersaturation was quantified by local elemental analysis using STEM-EDX mapping. Table 3 displays the chemical content (Al, Si, Mg) from two local regions, namely the α-Al cellular region and Si-rich eutectic network for the respective conditions (AB, DA, and SR). Figure A1 in the appendix displays the selection of areas used for the local chemical measurement. As the information collected from the STEM-EDX measurement originates from an ultra-thin sample (~ 100 nm), not the absolute concentration values but rather their relative comparison and how they evolve during the different heat treatments should be considered. In the AB condition, 2.7 wt% Si and 0.5 wt% Mg are in solid solution with Al, as indicated by the chemical measurement within the α-Al cellular region. Both Si and Mg solute concentrations decrease significantly for the two heat-treated conditions (DA, SR). Besides this, the Si-rich eutectic network shows a significant enrichment of Si (29.6 wt%) and Mg (1.7 wt%) compared to the primary Al cell.

**Table 3:** Chemical elemental quantification (Al, Si, Mg) based on local STEM-EDX mappings from the α-Al cellular region and Al/Si eutectic network in the MPC.

Local region	Condition	Al [wt%]	Si [wt%]	Mg [wt%]
α-Al cell in MPC	AB	96.8 ± 3.0	2.7 ± 0.2	0.5 ± 0.1
	DA	98.9 ± 3.2	0.7 ± 0.1	0.4 ± 0.1
	SR	99.5 ± 3.3	0.3 ± 0.1	0.1 ± 0.02
Eutectic network in MPC	AB	68.7 ± 2.3	29.6 ± 0.7	1.7 ± 0.2
	DA	70.4 ± 2.3	27.7 ± 0.5	1.9 ± 0.2
	SR	Not applicable due to disintegration of the eutectic network		

The standard error represents the standard deviation based on four measurement points per condition.

## Discussion

### Solidification microstructure

Despite the rapid solidification, the as-printed bulk mesostructure exhibits a microstructural distinction between the MPC, MPB, and HAZ (Fig. 4). This transition in microstructure is, on the one hand, induced by the local variation in cooling rate, spatial thermal gradient (G $\left[\frac{K}{m}\right]$) and growth rate (R $\left[\frac{m}{s}\right]$) within the meltpool^19–22^, and on the other hand, induced by the cyclic thermal history across multiple layers^23,24^. Literature^23,24^ already contains detailed computational parametric studies, describing the thermal behaviour of multi-layered LPBF-processed AlSi10Mg. Therefore, this work will focus on the discussion of the solidification microstructure within the meltpool.

#### Variation of cooling rate and cell size within the meltpool

Matyja et al. report a semi-empirical relationship between the primary dendritic arm spacing (*PDAS, [m]*) and the cooling rate (*G× R,* $\left[\frac{K}{s}\right]$ ) for rapidly cooled Al-Si alloys and is given by Eq. (1)^25^:

1 $$PDAS=a{(G\times R)}^{-n}$$

with the Al-Si material constant, *a* = 43.2 $[m{\left(\frac{K}{s}\right)}^{n}]$, and the exponent, *n* = 0.324 $[-]$. Equation (1) allows predicting the local cooling rates of the MPC and MPB, based on the observed PDAS in the local regions, displayed in Table 4. The MPC exhibits primary cells with an average PDAS of 0.5 ± 0.1 µm, resulting in an estimated cooling rate of 9.5 × 10^5^ K/s. The MPB presents coarser cells with an average PDAS of 0.9 ± 0.2 µm, resulting in an estimated cooling rate of 1.6 × 10^5^ K/s. Besides these semi-empirical predictions, the local cooling rates of MPC and MPB were also predicted based on the single-track transient heat transfer COMSOL model with identical laser setting parameters as used in the LPBF process. The heat transfer model predicts a cooling rate of 8.3 × 10^5^ K/s in the MPC, compared to 4.4 × 10^5^ K/s in the MPB, as shown in Table 4. The cooling rate predictions from the semi-empirical model (Eq. 1) and the numerical heat transfer model confirm the same trend. Likewise, the measured cell sizes and predicted cooling rates show good agreement with other observations from literature^25,26^. Lastly, the HAZ is shortly exposed to annealing temperatures (400–550 °C) due to the characteristic cyclic heating and cooling pattern induced by the incremental layerwise building process. Thermal simulations on LPBF-processed AlSi10Mg from Hu et al.^23^ and Li et al.^24^ confirm that the HAZ is exposed to this annealing temperature range. This local annealing exposure results in partial disintegration and spheroidisation of the Si-rich eutectic network in the HAZ.

**Table 4:** Cell size quantification of the MPC and MPB, and the estimation of local cooling rates based on a semi-empirical equation and numerical model.

Zone	PDAS [µm]	Semi-empirical model cooling rate^a^ [10^5^ K/s]	Numerical model cooling rate^b^ [10^5^ K/s]
MPC	0.5 ± 0.1	9.5	8.3 ± 2.0
MPB	0.9 ± 0.2	1.6	4.4 ± 0.5

^a^Cooling rate prediction based on the analytical equation Eq. (1).

^b^Cooling rate prediction based on the numerical heat transfer model.

#### Solidification morphologies within the meltpool

**Figure 6:**
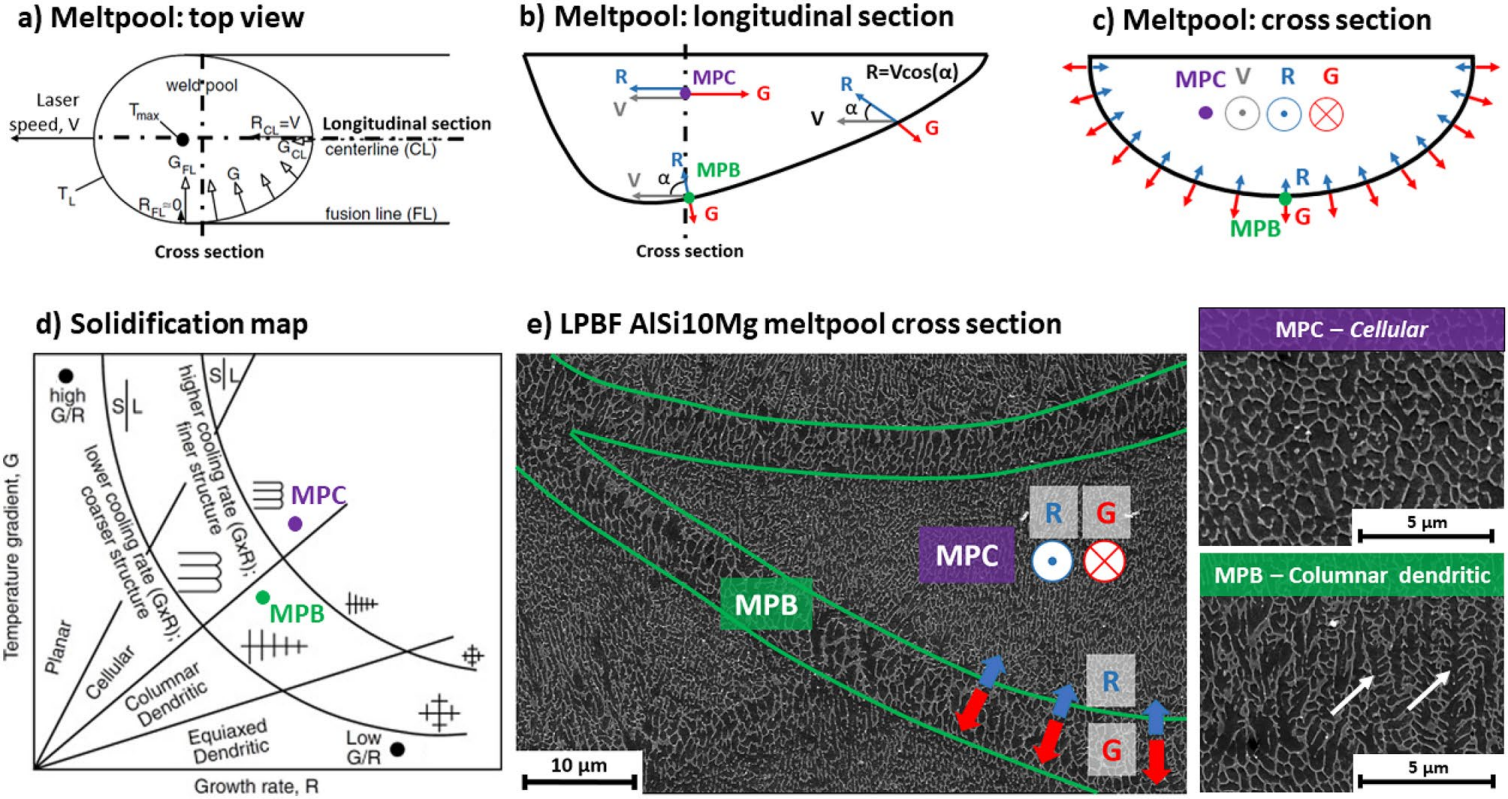
Metlpool schematics indicating local principal thermal gradient vectors (G) and interface growth rate vectors (R) within the meltpool, affecting the solidification modes in the meltpool centre (MPC) and meltpool boundary (MPB). (**a**) meltpool top view; (**b**) meltpool longitudinal section; (**c**) meltpool cross-section; (**d**) solidification map; (**e**) SEM image of LPBF AlSi10Mg (AB) showing the microstructure within the meltpool with a cellular morphology at the MPC and columnar dendritic morphology at the MPB. (**a**) and (**d**) adopted from Kou^20^.

Al-Si alloys obtain their irregular eutectic microstructures from the uncoupled solidification, where the α-Al matrix (non-faceted crystal) and the reinforced Si phase (faceted crystal) grow in a loosely coordinated manner from the liquid phase^19^. For hypo-eutectic Al-Si alloys (Si < 12.6 wt%), the solid/liquid (S/L) interface is predominantly controlled by the non-faceted α-Al matrix, which in turn is easily affected by the local solidification conditions (i.e. thermal gradient, growth rate)^19^. Authors^20–22,27,28^ have well established the rapid solidification behaviour of alloys processed via laser-based consolidation. Mohammadpour et al.^21^ discuss a reassessment of the solidification map for LPBF Al–Si–Mg alloys and explain how a variation in thermal gradient (G) and S/L interface growth rate (R) can affect the solidification modes in the meltpool. The solidification behaviour of LPBF AlSi10Mg within the meltpool is briefly discussed here. As support for this discussion, Fig. 6a–c displays schematics of the local solidification conditions (G, R) in the meltpool, while Fig. 6d,e indicates the correlation between these local solidification conditions (G, R) and the observed microstructural features within the meltpool. During the LPBF solidification, the thermal gradient vector (G) and S/L growth rate vector (R) vary in direction and magnitude within the meltpool (MPC, MPB). In the MPC region, high cooling rates apply, and the principal G and R vectors are oriented along the direction of the scan track, as displayed in Fig. 6a–c. There, the growth rate vector (R) reaches its maximum magnitude approaching the laser scanning speed^22^. As such, the local solidification in the MPC results in a fine cellular morphology, indicated in Fig. 6d,e. On the other hand, in the MPB region, lower cooling rates apply and the heat extraction at the MPB is more homogeneous due to the heat sink effect of the solidified substrate, surrounding the MPB. Therefore, multi-directional thermal gradient and growth rate vectors apply locally at the MPB, as shown in Fig. 6a–c. The G and R vectors are lower in magnitude compared to those in the MPC. Consequently, a coarser microstructure is obtained, and a transition in solidification morphology evolving from cellular features in the MPC towards more pronounced columnar dendritic solidification features in the MPB region. The white arrows indicate this morphological transition in Fig. 6e. The observations mentioned above and discussion are in agreement with the solidification behaviour of rapidly solidified eutectic alloys^19,22,27,29^.

#### Microsegregation resulting in the formation of a cellular solidification morphology

The STEM-EDX mappings, displayed in Fig. 5c,f,i, and the local chemical quantification (Table 3) show proof of the Si microsegregation at the primary α-Al cell boundaries in the AB microstructure. Microsegregation is associated with a local fluctuation in chemical composition caused by an unstable solidification interface, which results from solute rejection and entrapment mechanisms during solidification^19^. Depending on the solidification conditions (G/R), this unstable solidification front can result in cellular or dendritic microstructural features^19^. In literature, the Scheil model is often used to describe the non-equilibrium state of LPBF as-built alloys^1,7,20,30^. However, due to the Scheil's assumptions that (I) no solid-state diffusion occurs and (II) a stable planar solid/liquid solidification front applies during Scheil solidification, the model is limited to predicting macrosegregation and fails to describe solidification features, such as cells dendrites, resulting from microsegregation^19,31,32^. On the other hand, Brody-Flemings' analytical microsegregation model^33^ proposes a more general form to describe the solid-state concentration. In contrast to Scheil's model, the Brody-Flemings microsegregation model is not restricted to a stable solidification front. Therefore, Brody-Flemings' model succeeds in describing the relation between local solid-state concentration and the solidification features caused by microsegregation, such as cells and dendrites^19,31^. This work aims to verify whether the Brody-Flemings cellular microsegregation model still applies to rapidly solidified AlSi10Mg and whether it can predict the observed cellular eutectic morphology in the MPC based on local Si solute concentration in the Al cell. Brody-Flemings cellular microsegregation model relies on the solid-state concentration profile, *C*_*s*_ $[-]$, given by Eq. (2):

2 $${C}_{s}={k}_{0}{C}_{0}\left[\frac{a}{{k}_{0}-1}+\left(1-\frac{a{k}_{0}}{{k}_{0}-1}\right){\left(1-{f}_{s}\right)}^{{k}_{0}-1}\right]$$

with the equilibrium partition coefficient, *k*_*0*_ $[-]$, the alloy solute concentration, *C*_*0*_ $[-]$, the cellular microsegregation parameter, *a* $[-]$, and the solid fraction, *f*_*s*_ $[-]$. The cellular microsegregation parameter, *a*, is given by Eq. (3):

3 $$a=\frac{G}{R} \cdot \frac{{D}_{l}}{{m}_{l}{C}_{0}}$$

with the spatial thermal gradient, *G* $\left[\frac{K}{m}\right]$, the solidification rate, *R* $\left[\frac{m}{s}\right]$, the diffusion coefficient in the liquid, *D*_*l*_ $\left[\frac{m^{2}}{s}\right]$, the liquidus slope, *m*_*l*_ $\left[K\right]$. As such, the cellular microsegregation parameter is determined by the solidification process condition (*G, R*) and the alloy characteristics (*D*_*l*_*, m*_*l*_*, C*_*0*_). Table A2 in appendix summarises the possible solidification modes depending on the regime of the microsegregation parameter and the partition coefficient, as described by the Brody-Flemings model*.* In case $\left|a\right|<\left|\frac{{k}_{0}-1}{{k}_{0}}\right|$ and ${k}_{0}<k<1$, an unstable solidification front is formed inducing microsegregation and resulting in a cellular morphology^19^. Under rapid solidification conditions, the partition coefficient (*k*) deviates from the equilibrium partition coefficient, *k*_*0*_^19,27,29^. Brody-Flemings cellular microsegregation model describes how the cellular partition coefficient, k_cell_, can be related to the solute concentration at the solid cellular tip, *C*_*s*_^*cell tip*^, as given by Eq. (4). Next, the cellular microsegregation parameter can then be calculated based on the cellular partition coefficient, as given by Eq. (5)^19^.

4 $${k}_{cell}=\frac{{C}_{S}^{cell tip} \, }{{C}_{0}}={k}_{0}\frac{{C}_{l}^{cell tip} \, }{{C}_{0}}$$

5 $$a=1-\frac{{k}_{cell}}{{k}_{0}}$$

Applying these equations to the Si solute concentration in the α-Al cell, measured by STEM-EDX, allows for estimating the microsegregation parameter. The outcome is summarised in Table 5 and indicates that the criterion to obtain a cellular solidification front (i.e. $\left|a\right|<\left|\frac{{k}_{0}-1}{{k}_{0}}\right|$) applies. As such, the formation of a cellular solidification morphology is confirmed. In summary, the Brody-Flemings cellular microsegregation model can predict the observed cellular solidification morphology of LPBF-processed AlSi10Mg based on the local Si solute concentration in the Al cell, measured by STEM-EDX.

**Table 5:** Estimation of microsegregation parameter, a, based on local Si solid solution content in the α-Al cellular region ($C_{Si_S}^{cell}$) measured by STEM-EDX.

AlSi10Mg	$C_{Si_S}^{cell}$[Si wt%]	${C}_{Si}$[Si wt%]	k_cell_ [–]	k_0_ [–]	$\frac{{k}_{0}-1}{{k}_{0}}$[–]	a [–]
α-Al cell in MPC	2.7	10	0.27	0.13	− 6.69	− 1.08

The equilibrium partition coefficient for Al-Si alloys is ${k}_{0}=0.13$ ^19^.

#### Solidification trajectory

**Figure 7:**
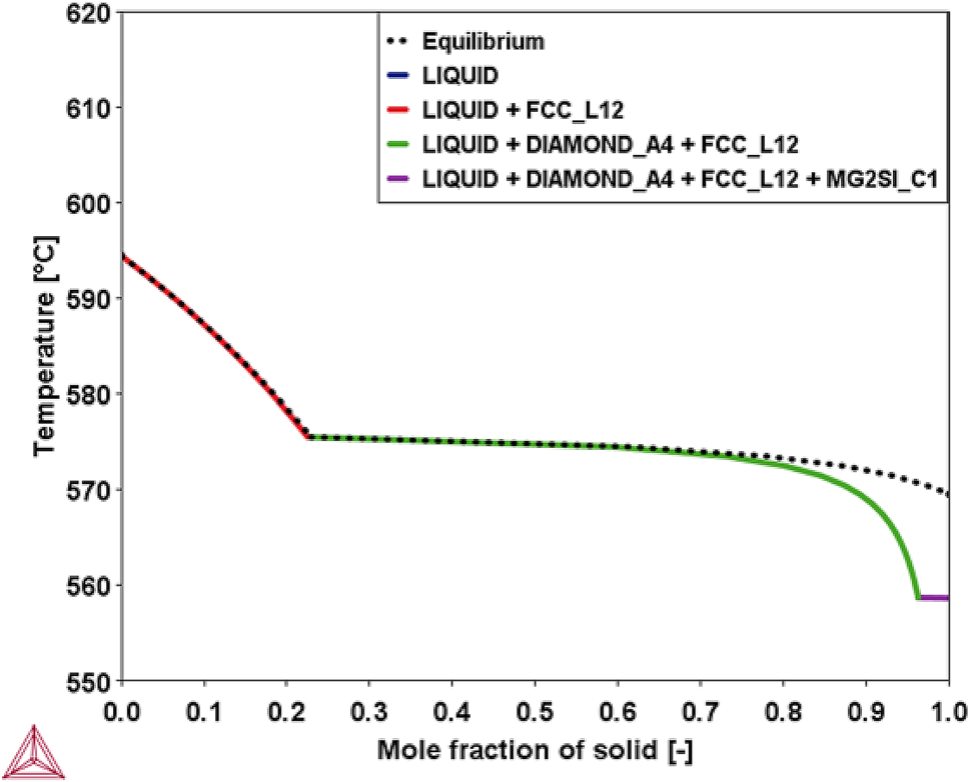
AlSi_10_Mg_0.3_ solidification trajectory under equilibrium and Scheil solidification condition is simulated in Thermo-Calc. Phases included in the model are liquid, primary Al (FCC_L12), Si (DIAMOND_A4), Mg_2_Si (MG2SI_C1).

**Figure 8:**
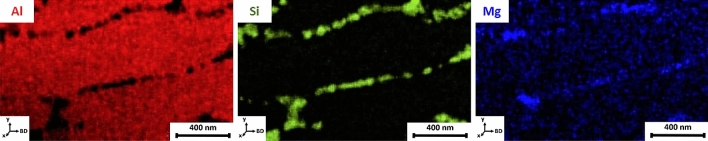
STEM-EDX mappings (Al, Si, Mg) representing a primary Al cell recorded at the MPC in the as-built LPBF-processed AlSi_10_Mg_0.3_ microstructure.

The solidification trajectory of AlSi10Mg0.3 under equilibrium and Scheil solidification conditions is calculated with Thermo-Calc, as shown in Fig. 7. The solute concentration profiles during the solidification trajectory are displayed in appendix Fig. A2. The equilibrium and Scheil solute concentrations (Si, Mg) in the primary Al phase and the last fraction of the liquid phase are summarised in Table 6 and compared with the solute concentrations collected by STEM-EDX from the respective primary α-Al cell and Al-Si eutectic network of the AB bulk microstructure. Both equilibrium and Scheil solidification models show significant enrichment of Si and Mg in the last liquid fraction. Following the solidification trajectory, the eutectic network can be considered representative for the last liquid fraction. A similar trend is observed in the LPBF as-built microstructure, where STEM-EDX measurements show significant enrichment of Si (29.6 wt%) and Mg (1.7 wt%) in the eutectic network at the Al cell boundaries. The Si and Mg enriched eutectic network is shown qualitatively in Fig. 8 and quantitatively in Table 3. Indeed, the solutes' equilibrium partition coefficients (k_0_ = 0.116 for Si, k_0_ = 0.195 for Mg) are much smaller than one. Therefore, the solutes will be rejected from the solidifying primary Al cells, thus enriching the remaining liquid fraction^26^.

**Table 6:** Si and Mg solute concentrations in α-Al cell^a^ and Al-Si eutectic network^b^ based on numerical equilibrium and Scheil models (Thermo-Calc) and experimental chemical analysis of the AB microstructure by STEM-EDX.

Solute concentration	MPC	Equilibrium solidification	Scheil solidification	STEM-EDX AB
Si	α-Al cell^a^	1.0–1.6	1.2–1.5	2.7 ± 0.2
	Eutectic network^b^	10.0–12.5	10.0–12.5	29.6 ± 0.7
Mg	α-Al cell^a^	0.3	0.05	0.5 ± 0.1
	Eutectic network^b^	0.3	0.4–4.5	1.7 ± 0.2

^a^Solute concentration (Si, Mg) from equilibrium and Scheil model are based on simulated solute concentrations in primary Al (FCC_L12).

^b^Solute concentration (Si, Mg) from equilibrium and Scheil model are based on simulated solute concentration in the last fraction of the liquid phase.

**Figure 9:**
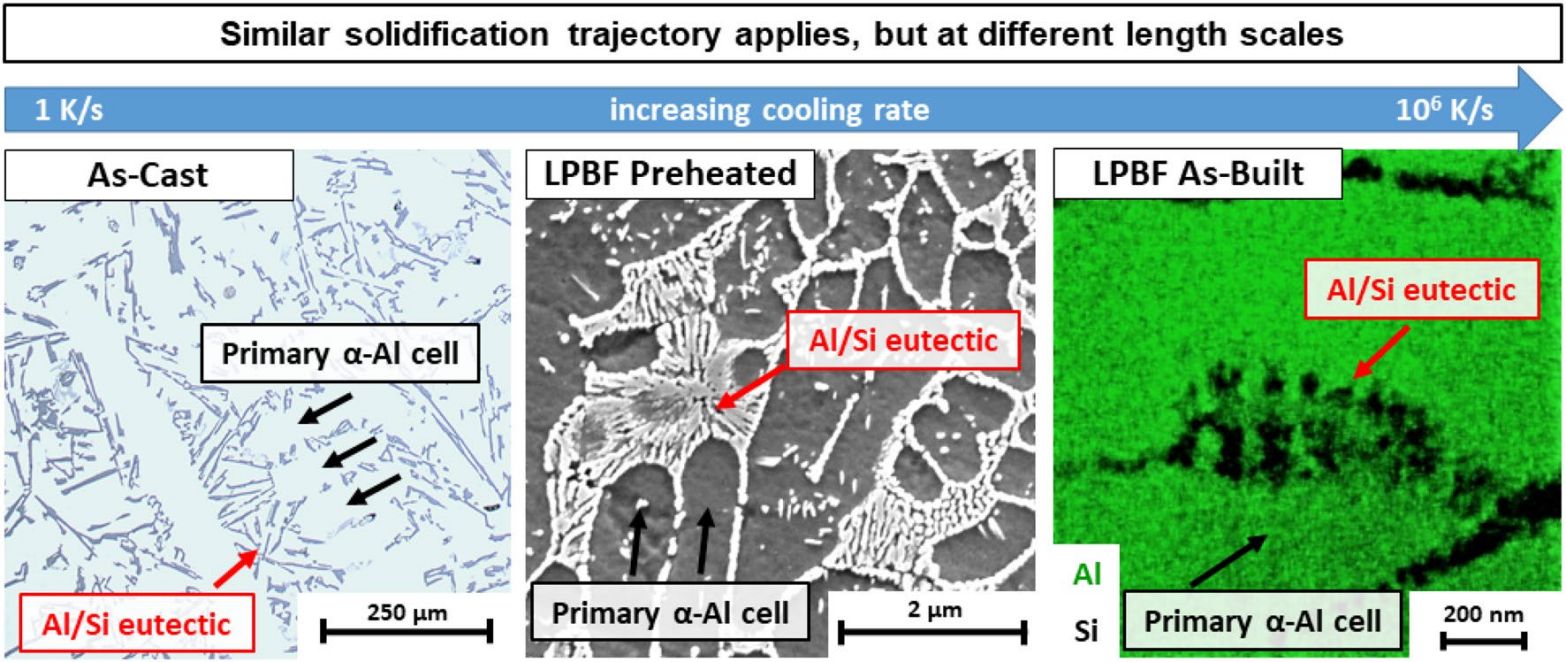
Different AlSi10Mg microstructures covering a broad solidification spectrum with cooling rates ranging from 1 K/s to 10^6^ K/s: As-cast (left), LPBF-processed with baseplate preheating at 200 °C (PH) (middle), LPBF As-built (AB) (right). The microstructural images indicate the microstructural scale effect and the observation of a repetitive microstructural pattern, as indicated by the arrows.

On the other hand, the rapid solidification during LPBF processing will cause entrapment of solute atoms in the primary Al cells. Both the equilibrium and Scheil solidification models underestimate the Si and Mg solute concentration in the primary Al cells compared to the STEM-EDX measurement (Si: 2.7 wt%, Mg: 0.5 wt%). Namely, a high degree of Si supersaturation (2.7 wt%) is observed in the primary Al cells based on these STEM-EDX measurements (Table 3), thus exceeding the maximum Si equilibrium solubility (1.65 wt%) at the eutectic temperature. Likewise, Zhao et al. performed local STEM-EDX measurements on as-built LPBF-processed AlSi10Mg and reported a similar Si solute content (2.2 at%) in the primary Al cell^34^. Besides the degree of supersaturation, this work shows that the predicted solidification trajectory seems to be retained under rapid solidification conditions. Hence, several AlSi10Mg microstructures, covering a broad spectrum of solidification conditions, ranging from casting (AC) to rapid solidification (PH, AB), are compared with each other, as displayed in Fig. 9. These microstructural images indicate a significant microstructural refinement when comparing rapid solidification with casting, thus confirming the well-established relation between the cooling rate and cell size, as given by Eq. (1).

Furthermore, at the triple point of the primary Al cells, a repetitive microstructural eutectic feature pattern is observed at different length scales when comparing the microstructures obtained through solidification at different cooling rates, ranging from 1 K/s (AC) up to 10^6^ K/s (LPBF AB). This observation confirms the fact that the solidification trajectory remains valid under rapid solidification conditions. The arrows in Fig. 9 show the eutectic structure that forms at the end of solidification for the respective solidification conditions and concomitant cooling rates. In summary, the high thermodynamic stability of eutectic alloys and the invariant eutectic phase transformation at the end of the solidification trajectory, result in the formation of a natural in-situ Al-Si composite arrangement. Hence, thanks to this intrinsic solidification behaviour, eutectic alloys adapt well to a broad spectrum of solidification conditions and cooling rates, ranging from casting to rapid solidification. Therefore, an increasing cooling rate leads to a microstructural refinement without fundamentally affecting the microstructural arrangement of the AlSi10Mg alloy. It is important to note that an increasing cooling rate enhances the Si solute supersaturation in the primary Al cell significantly, yet it does not appear to affect the phase stability or the S/L interface stability.

### Effect of heat treatment on microstructure and properties

Table 7 summarises the threefold interrelationship between the processing conditions, the microstructural features, and the macroscopic material properties for the respective conditions: AB, DA, SR, and PH. A detailed discussion of the critical process parameters (LPBF and heat treatment) determining the microstructural features at different length scales (macro-, meso-, micro-, and nano-scale) and the concomitant macroscopic material properties (tensile and thermal properties), is covered in the following sections. Each row in Table 7 displays the predominant microstructural feature affecting the particular macroscopic material property.

**Table 7:** Process-microstructure-macroscopic material property table with the critical process parameters (LPBF, HT) affecting the microstructural features and concomitant mechanical and thermal properties.

	Process critical parameter	Microstructural features	Macroscopic material properties
AB	LPBF: scan speed (cooling rate)	Al-matrix: ultrafine cellular Al/Si eutectic: continuous network	Strength (UTS, YS): ++ Elongation: −
		Supersaturation: 2.7 wt% Si	Conductivity: −
		Mesostructure: heterogeneous → MPC/MPB/HAZ	Elastic isotropy: ++ Plastic isotropy: −−
DA	HT: minimum holding time	Al-matrix: ultrafine cellular + acicular Si precipitates Al/Si eutectic: continuous network	Strength (UTS, YS): ++ Elongation: −−
		Supersaturation: 0.7 wt% Si	Conductivity: +
		Mesostructure: heterogeneous → MPC/MPB/HAZ	Elastic isotropy: ++ Plastic isotropy: −−
SR	HT: temperature	Al-matrix: ultrafine cellular + fine Si precipitates Al/Si eutectic: disintegrated + coarse Si precipitates	Strength (UTS, YS): + Elongation: ++
		Supersaturation: 0.3 wt% Si	Conductivity: ++
		Mesostructure: heterogeneous → MPC/MPB/HAZ Less dense bi-modal distribution of Si precipitates	Elastic isotropy: ++ Plastic isotropy: −
PH	LPBF: preheating temperature	Al-matrix: coarse cellular Al/Si eutectic: continuous network	Strength (UTS, YS): + Elongation: −
		Supersaturation: 0.2 wt% Si	Conductivity: ++
		Mesostructure: heterogeneous → MPC/MPB/HAZ	Elastic isotropy: ++ Plastic isotropy: −

#### Si supersaturation in the as-built state

**Figure 10:**
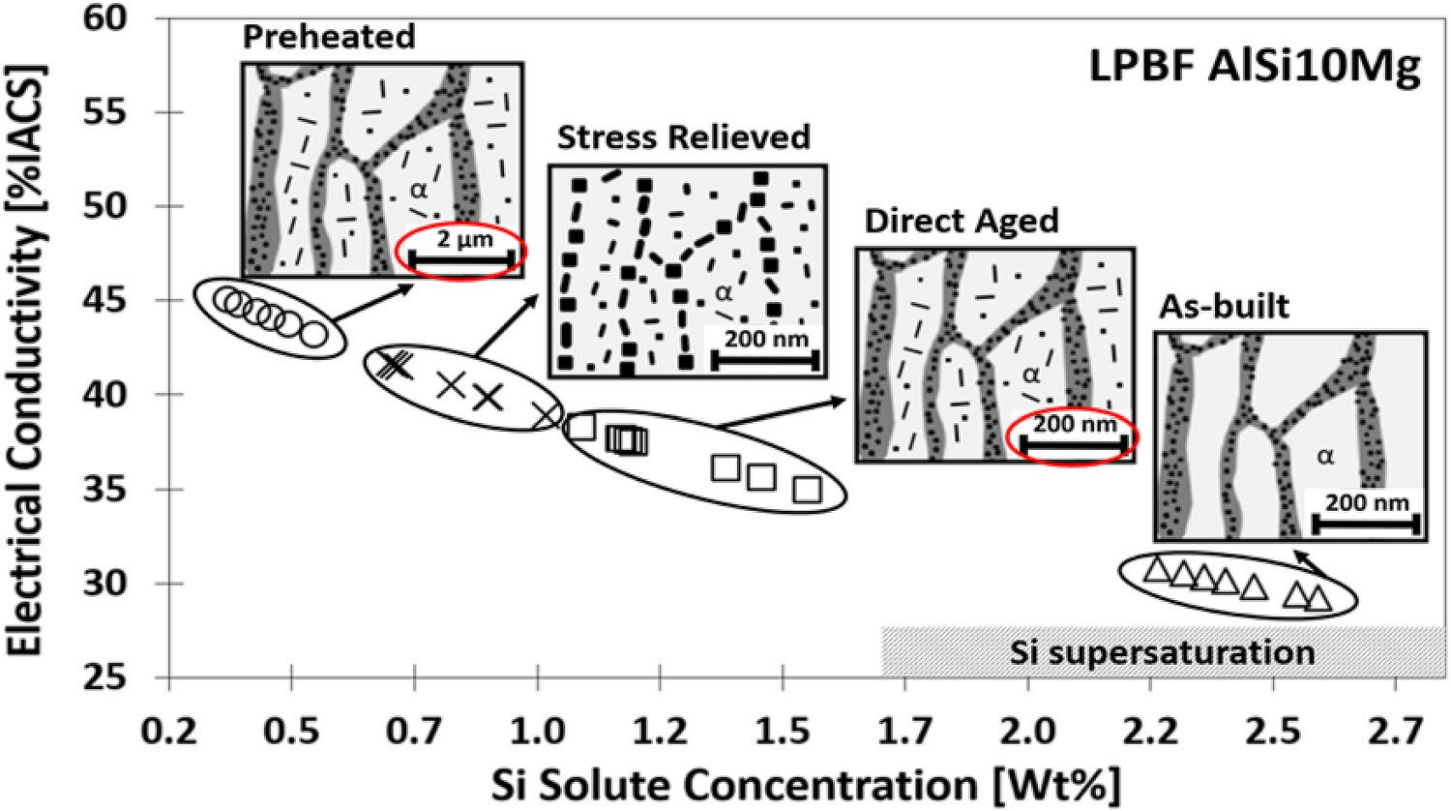
LPBF-processed AlSi10Mg: Schematic microstructural evolution upon heat treatment and its correlation with the predicted Si solute concentration in the Al matrix as a function of the electrical conductivity.

Rapid solidification enhances the solid solute concentration in the matrix^29,30,35–37^. The degree of Si supersaturation drives the Si precipitation, thus driving the microstructural evolution during heat treatment of LPBF-processed AlSi10Mg^10,11,26,38^. Therefore, it is critical to monitor the evolution of Si supersaturation in the Al matrix. The Si supersaturation can be estimated based on electrical resistivity (ER) measurements^39–41^. Tang et al. report a semi-empirical equation given by Eq. (6), to estimate the Si solute concentration in Al-Si alloys^26^.

6 $${W}_{Si _S}=\frac{185}{\sigma }-2.84-0.094{W}_{Si}$$

with the weight fraction of Si in solid solution, ${W}_{Si _S}$ $[wt\%]$*,* the electrical conductivity relative to the International Annealed Copper Standard *σ* $[\%IACS]$, and the total Si weight fraction of the AlSi10Mg alloy, *W*_*Si*_ $[wt\%]$. It is important to note that the current electrical conductivity model neglects the influence of Mg on the electrical conductivity of AlSi10Mg0.3. The microstructural evolution and the concomitant Si solute concentration in the Al matrix is displayed in Fig. 10 and Table 7 for the respective LPBF-processed AlSi10Mg conditions. The estimated Si solute concentration estimated from the electrical conductivity model shows good agreement with the STEM-EDX Si concentration in the α-Al cell of the MPC (Table 3) for the respective conditions (AB, DA, SR). The AB condition shows a Si solute concentration ranging from 2.0 to 2.7 wt%, thus exceeding the maximum equilibrium Si solid solubility in Al (1.65 wt%). Zhao et al.^34^ and Maeshima et al.^30^ report similar Si solute concentration (2.2 at%) based on STEM-EDX. The Si solute concentrations measured from ER and STEM-EDX confirm that the rapid cooling (10^4^–10^6^ K/s) induces a significant enhancement of the Si solid solubility in LPBF-processed AlSi10Mg. Upon heat treatment, the Si solute concentration drastically decreases with increasing heat treatment temperature: DA (1.0–1.5 wt% Si) and SR (0.65–1.0 wt% Si). The correlation between the Si solute concentration and the microstructural evolution, shown in Fig. 10, proofs that the Si supersaturation drives the Si precipitation in the Al-matrix. Next, LPBF processing of AlSi10Mg with baseplate preheating (PH) attains the lowest Si solute concentration (0.3–0.5 wt%). This low solute concentration could imply that significant lower solidification rates apply when processing with baseplate preheating at 200 °C. However, it is important to mention that the in-situ ageing effect, reducing the Si solute concentration, cannot be neglected in the latter case.

To conclude, the electrical conductivity model can predict the Si solute concentration in AlSi10Mg. This model allows monitoring the extent of Si supersaturation and unravels how the Si supersaturation contributes to the microstructural evolution during heat treatment. Therefore, electrical conductivity models can be used for LPBF process optimisation and heat treatment optimisation of precipitation-hardenable alloys.

#### Microstructural evolution during heat treatment

DSC and ER measurements were combined with microstructural analysis and STEM-EDX to accurately map the relation between the Si supersaturation and the microstructural evolution of LPBF-processed AlSi10Mg during heat treatment. The Si supersaturated-state of the AB microstructure, shown in Fig. 5a–c, drives the Si precipitation during heat treatment. The exothermic DSC reaction peak (1) from 195 to 205 °C, shown in Fig. 3a, is attributed to the Si precipitation from the supersaturated Al cell. Furthermore, the disappearance of this DSC peak in the second DSC cycle (i.e. annealed condition), given in Fig. 3b, confirms the irreversible process of the Si supersaturation driving the Si precipitation during heat treatment. Indeed, the same Si precipitation reaction occurs during DA. The DA microstructure, shown in Fig. 5d–f, exhibits an ultrafine distribution of Si acicular and cubic-like Si precipitates in the primary Al cell, while the Si-rich eutectic network remains intact. These observations are summarised in Table 7 and agree with literature^6,7,34,38,42^.

Next, during a stress relief (SR) heat treatment, the Si supersaturation further decreases due to the intracellular Si precipitation. Moreover, the Si-eutectic network starts to disintegrate, and individual, spherically-shaped Si particles, lying along the Al cell boundaries, as schematically shown in Fig. 10, start to appear. The increased Si diffusional activity drives this disintegration and spheroidisation and represents the second exothermic DSC peak (305–320 °C), as indicated in Fig. 3a. Similar observations were reported by Fiocchi et al.^10^. Likewise, the Si diffusional activity allows Ostwald ripening of the fine intracellular Si precipitates. Thus, the SR microstructure evolves into a less dense and bimodal distribution of spherical Si precipitates, as displayed in Fig. 5g–i, and summarised in Table 7. Likewise, Zhao et al. report a bimodal Si particle distribution for similar HT conditions^34^. The disintegration of the eutectic network and the Ostwald ripening of the fine Si precipitates are irreversible transformation processes and explain the disappearance of DSC peak (2) in the annealed condition, shown in Fig. 3b. Despite the disintegration of the eutectic network and spheroidisation of Si precipitates during SR, the mesostructure has not entirely homogenised, as shown in Fig. 5g. Indeed, the MPB remains apparent in the mesostructure after SR. Next, solution annealing at high temperatures (400–550 °C) will dissolve the Si precipitates again in the Al matrix due to the increasing Si solubility, and is represented by the endothermic DSC peak (350–550 °C), shown in Fig. 3a,b. Finally, the reversible exothermic DSC peaks (4) and (5) during cooling could be attributed to the precipitation of Si and Mg_2_Si, respectively. The gradual decrease of the Si and Mg solubility in Al during cooling drives these precipitation reactions. Based on the insights gained from the Si supersaturation and the microstructural evolution of LPBF-processed AlSi10Mg during heat treatment, it can be concluded that the solution annealing and quenching step from the T6 heat treatment have become redundant for thermal processing of LPBF-processed AlSi10Mg.

#### Mechanical response variation induced by heat treatments

The correlation between the microstructural features and evolution during heat treatment and their concomitant mechanical response is summarised in Table 7. This paragraph covers a more detailed discussion of this correlation. The ultrafine microstructure, together with the natural in-situ Al/Si composite structure, are dominant contributors to the high strength properties of the AlSi10Mg alloy in AB condition. Consensus exists in literature about the Si-rich continuous eutectic network acting as the main load-bearing structure and being a barrier for dislocation movement^34,43,44^. Therefore, a significant strain hardening effect is observed for the AB and DA conditions, which exhibit a continuous Si-rich eutectic network. Moreover, the Si supersaturation contributes to a solution-strengthening effect in the AB material. On the other hand, the precipitation of ultrafine acicular and cubic-like Si particles in the DA microstructure act as additional dislocation movement barriers. Therefore, the highest yield strength among all conditions, yet with impaired ductility, is obtained in the DA condition. The SR microstructure contains a disintegrated and spheroidised Si-rich eutectic network, and a coarsening of the intracellular Si precipitates occurs. This microstructural evolution results in a significant strength loss and a notable increase in ductility. Moreover, it results in a significant decrease in strain hardening. Lastly, the preheated condition (PH) shows a similar, yet coarser microstructure compared to the one observed in the DA sample, as schematically indicated in Fig. 10. The reduced cooling rates in LPBF processing with baseplate preheating cause this coarsening effect. In general, the Si phase morphology and size in the intracellular and intercellular regions are the major contributing microstructural features affecting the mechanical properties^6,7,42^. In summary, the tailored heat treatments for LPBF-processed AlSi10Mg (DA, SR, PH), offer an interesting range of excellent mechanical and thermal properties.

### Elastic and plastic anisotropic behaviour

Regardless of the LPBF-processed and heat-treated conditions (AB, DA, SR), Figs. 1 and 2 show a notable higher ductility for the horizontal samples (XY) when compared to their respective vertical tensile samples (Z). On the other hand, the vertical and horizontal tensile coupons show almost similar yield strength (YS) and ultimate tensile strength (UTS) values per condition (AB, DA, SR). Based on the RUS characterisation, the elastic anisotropy factors show values close to unity (≈1.04) for the respective conditions (AB, DA, SR), thus confirming the isotropic elastic behaviour of the LPBF-processed material^45^. Nonetheless, the mechanical anisotropic behaviour of LPBF-processed Al-Si alloys has been reported in literature^8,9,26,34,46–50^. Recent investigations claim that LPBF-processed Al-Si alloys exhibit isotropic elastic behaviour, followed by an anisotropic plastic deformation behaviour^8,26,34,46,48^. The experimental observations from this research work support this hypothesis. Consensus exists concerning the root cause of the anisotropic plastic behaviour. It appears that the coarse microstructure in the MPB and the disintegrated Si-rich eutectic network in the HAZ act as a weak zone compared to the ultrafine Al cells with continuous Si-rich eutectic network in the MPC. Therefore, the MPB and HAZ regions are more prone to plastic deformation. Consequently, damage initiation occurs along the MPB and HAZ due to debonding and cavity nucleation at the Al/Si interface, followed by coalescence of the deformation micro-cavities, resulting in macro-crack formation^34,47^. Hence, the orientation of the meltpool relative to the tensile loading direction induces the plastic anisotropy observed in LPBF-processed Al-Si alloys^8,26,34,46,48^. The plastic anisotropy of the SR condition is reduced significantly compared to the AB and DA condition, but cannot be considered fully isotropic. Namely, despite the disintegration of the Al/Si eutectic network and spheroidisation of Si precipitates, the mesostructure has not entirely homogenised, as shown in Fig. 5g. Therefore, the MPB is still apparent after SR and remains a weak region for plastic deformation. To conclude, the plastic anisotropic behaviour originates from the heterogeneous mesostructure across the multiple layers.

## Conclusion

A comprehensive study was conducted on LPBF-processed and heat-treated AlSi10Mg, covering a discussion on rapid solidification behaviour and unravelling the multi-scale structure–property relationship. This research confirms that a similar solidification trajectory applies for mould-cast and LPBF-processed AlSi10Mg, but at different length scales. The observed similarity in solidification behaviour triggers the reason why the Brody-Flemings microsegregation solidification model was able to predict the cellular morphology of the LPBF as-printed microstructure. Despite the similar solidification trajectory, the rapid solidification (LPBF) of AlSi10Mg occurs at a much finer length scale. Therefore, the LPBF microstructure exhibits a significant grain refinement and a high degree of Si supersaturation, when compared to its cast peer. The grain refinement and Si supersaturation are critical assets of the as-printed microstructure, and play a vital role in achieving superior mechanical and thermal properties during post heat treatment. The high-temperature annealing step of a conventional T6 heat treatment eliminates these two critical assets, and thus vanishes the superior strengthening potential of the LPBF-processed AlSi10Mg microstructure. Therefore, a T6 heat treatment for LPBF AlSi10Mg should only be considered for rapid prototyping purposes. Next, this work covers a process-structure–property table, summarising the critical process parameters (LPBF, heat treatment) affecting the microstructural features at different length scales (macro-, meso-, micro-, and nano-scale), and their concomitant mechanical and thermal properties. Lastly, an electrical conductivity model was able to predict the Si solute concentration in Al. This model allows monitoring and understanding the Si supersaturation and its driving force for microstructural evolution. Such an electrical conductivity model can be proposed for LPBF process optimisation and heat treatment optimisation of precipitation-hardenable alloys. The insights gained from this profound experimental work could be used to verify computational models simulating the microstructural evolution of LPBF-processed and post heat-treated AlSi10Mg.

## Supplementary Information

Supplementary Information.

## References

[1] DebRoy T (2018). Additive manufacturing of metallic components—Process, structure and properties. Prog. Mater. Sci..

[2] Li N (2018). Progress in additive manufacturing on new materials: A review. J. Mater. Sci. Technol..

[3] de Formanoir C (2020). Increasing the productivity of laser powder bed fusion: Influence of the hull-bulk strategy on part quality, microstructure and mechanical performance of Ti-6Al-4V. Addit. Manuf..

[4] Metelkova J, Ordnung D, Kinds Y, Witvrouw A, van Hooreweder B (2020). Improving the quality of up-facing inclined surfaces in laser powder bed fusion of metals using a dual laser setup. Procedia CIRP.

[5] Aversa A (2019). Aluminum alloys specifically designed for new aluminum alloys specifically designed for laser powder bed fusion: A review laser powder bed fusion: a review abstract. Materials.

[6] Li W (2016). Effect of heat treatment on AlSi10Mg alloy fabricated by selective laser melting: Microstructure evolution, mechanical properties and fracture mechanism. Mater. Sci. Eng. A.

[7] Takata N, Kodaira H, Sekizawa K, Suzuki A, Kobashi M (2017). Change in microstructure of selectively laser melted AlSi10Mg alloy with heat treatments. Mater. Sci. Eng. A.

[8] Buchbinder D (2015). Selective laser melting of aluminum die-cast alloy—Correlations between process parameters, solidification conditions, and resulting mechanical properties. J. Laser. Appl..

[9] Aboulkhair NT, Maskery I, Tuck C, Ashcroft I, Everitt NM (2016). The microstructure and mechanical properties of selectively laser melted AlSi10Mg: The effect of a conventional T6-like heat treatment. Mater. Sci. Eng. A.

[10] Fiocchi J, Tuissi A, Bassani P, Biffi CA (2017). Low temperature annealing dedicated to AlSi10Mg selective laser melting products. J. Alloys Compd..

[11] Van Cauwenbergh, P., Beckers, A., Lore, T., Van Hooreweder, B. & Vanmeensel, K. Heat treatment optimization via thermo-physical characterization of AlSi7Mg and AlSi10Mg manufactured by laser powder bed fusion. *Eur. Powder Metall. Assoc.* 1–7 (2018).

[12] DebRoy T (2019). Scientific, technological and economic issues in metal printing and their solutions. Nat. Mater..

[13] Collins PC, Brice DA, Samimi P, Ghamarian I, Fraser HL (2016). Microstructural control of additively manufactured metallic materials. Annu. Rev. Mater. Res..

[14] Ivekovic, A., Montero-Sistiaga, M. L., Vleugels, J., Kruth, J.-P. & Vanmeensel, K. Combined experimental numerical investigation of crack mitigation in SLM processed Ni-based superalloys (submitted for revision). *J. Mater. Process. Technol.*

[15] Sedlak, P. & Seiner, H. J. Z. Determination of All 21 Independent Elastic Coefficients of Generally Anisotropic Solids by Resonant Ultrasound Spectroscopy : Benchmark Examples. 1073–1085 (2014). 10.1007/s11340-014-9862-6

[16] Seiner H (2010). Application of ultrasonic methods to determine elastic anisotropy of polycrystalline copper processed by equal-channel angular pressing. Acta Mater..

[17] Jones W, March NH (1973). Theoretical solid state physics.

[18] Bergman TL, Lavine AS, Incropera FP (2011). Fundamentals of Heat and Mass Transfer.

[19] Glicksman ME (2011). Principles of Solidification.

[20] Kou S (2002). Welding Metallurgy.

[21] Mohammadpour P, Plotkowski A, Phillion AB (2020). Revisiting solidi fi cation microstructure selection maps in the frame of additive manufacturing. Addit. Manuf..

[22] Kurz W (2001). Solidification microstructure-processing maps: Theory and application. Adv. Eng. Mater..

[23] Hu H, Ding X, Wang L (2016). Numerical analysis of heat transfer during multi-layer selective laser melting of AlSi10Mg. Optik.

[24] Li Y, Gu D (2014). Parametric analysis of thermal behavior during selective laser melting additive manufacturing of aluminum alloy powder. Mater. Des..

[25] Matyja H (1968). The effect of cooling rate on the dendrite spacing in splat-cooled aluminium alloys. J. Inst. Met..

[26] Tang, M. Inclusions , Porosity, and Fatigue of AlSi10Mg Parts Produced by Selective Laser Melting (2017).

[27] Kurz, W. & Fisher, D. J. *Fundamentals of Solidification* (Trans Tech Publications Ltd., 1992).

[28] Gu D, Shi Q, Lin K, Xi L (2018). Microstructure and performance evolution and underlying thermal mechanisms of Ni-based parts fabricated by selective laser melting. Addit. Manuf..

[29] Pierantoni M, Gremaud M, Magning P, Stoll D, Kurz W (1992). The coupled zone of rapidly solidified Al-Si alloys in laser treatment. Acta Mater..

[30] Maeshima T, Oh-ishi K (2019). Solute clustering and supersaturated solid solution of AlSi10Mg alloy fabricated by selective laser melting. Heliyon.

[31] Shin YH, Kim MS, Oh KS, Yoon EP, Hong CP (2001). An analytical model of microsegregation in alloy solidification. ISIJ Int..

[32] Won YM, Thomas BG (2001). Simple model of microsegregation during solidification of steels. Metall. Mater. Trans. A Phys. Metall. Mater. Sci..

[33] Flemings MC (1974). Solidification Processing.

[34] Zhao L (2019). Damage mechanisms in selective laser melted AlSi10Mg under as built and different post-treatment conditions. Mater. Sci. Eng. A.

[35] Li XP (2015). A selective laser melting and solution heat treatment refined Al–12Si alloy with a controllable ultrafine eutectic microstructure and 25 % tensile ductility. Acta Mater..

[36] Sobolev SL (2015). Direct Driving force for binary alloy solidification under far from local equilibrium conditions. Acta Mater..

[37] Tang M, Pistorius PC, Narra S, Beuth JL (2016). Rapid solidification : Selective laser melting of AlSi10Mg. Jom.

[38] Marola S (2018). A comparison of selective laser melting with bulk rapid solidification of AlSi10Mg alloy. J. Alloys Compd..

[39] Hatch, J. E. Aluminum: properties and physical metallurgy. In *Aluminum: Properties and Physical Metallurgy* 204–205 (1984).

[40] Mulazimoglu MH, Drew RAL, Gruzleski JE (1989). Solution treatment study of cast Al–Si alloys by electrical conductivity. Can. Metall. Q..

[41] Mulazimoglu MH, Drew RAL, Gruzleski JE (1989). The electrical conductivity of cast Al−Si alloys in the range 2 to 12.6 wt pct silicon. Metall. Trans. A.

[42] Wei P (2017). The AlSi10Mg samples produced by selective laser melting: Single track, densification, microstructure and mechanical behavior. Appl. Surf. Sci..

[43] Chen B (2017). Strength and strain hardening of a selective laser melted AlSi10Mg alloy. Scr. Mater..

[44] Kim DK (2017). Evaluation of the stress-strain relationship of constituent phases in AlSi10Mg alloy produced by selective laser melting using crystal plasticity FEM. J. Alloys Compd..

[45] Newnham R (2005). Properties of Materials—Anisotropy, Symmetry, Structure.

[46] Zhang J, Song B, Wei Q, Bourell D, Shi Y (2018). A review of selective laser melting of aluminum alloys: Processing, microstructure, property and developing trends. J. Mater. Sci. Technol..

[47] Suryawanshi J (2016). Simultaneous enhancements of strength and toughness in an Al-12Si alloy synthesized using selective laser melting. Acta Mater..

[48] Yang KV, Rometsch P, Davies CH, Huang A, Wu X (2018). Effect of heat treatment on the microstructure and anisotropy in mechanical properties of A357 alloy produced by selective laser melting. Mater. Des..

[49] Rashid R (2018). Effect of energy per layer on the anisotropy of selective laser melted AlSi12 aluminium alloy. Addit. Manuf..

[50] Kempen K, Thijs L, Humbeeck JV, Kruth J (2012). Mechanical properties of AlSi10Mg produced by Selective Laser Melting. Phys. Procedia.

