# Supplementary material for: Unravelling the multi-scale structure–property relationship of laser powder bed fusion processed and heat-treated AlSi10Mg
Source: Sci Rep. 2021 Mar 19;11:6423. doi: 10.1038/s41598-021-85047-2 (PMC7979699; doi:10.1038/s41598-021-85047-2)
Supplement: Supplementary file 1 — Supplementary Information. [file 41598_2021_85047_MOESM1_ESM.docx]

**Unravelling the multiscale structure-property relationship of laser powder bed fusion processed and heat-treated AlSi10Mg**

# Authors

Van Cauwenbergh P.^1^, Samaee V.^2^, Thijs L.^1^, Nejezchlebová J.^3^, Sedlák P.^3^, Iveković A.^4^, Schryvers D.^2^, Van Hooreweder B.^5^, Vanmeensel K.* ^6^

^*^ Corresponding author

*^1^ 3D Systems Leuven, Grauwmeer 14, B-3001 Leuven, Belgium;
P. Van Cauwenbergh ([pierre.vancauwenbergh@3dsystems.com](mailto:pierre.vancauwenbergh@3dsystems.com)),
Dr. L. Thijs ([lore.thijs@3dsystems.com](mailto:lore.thijs@3dsystems.com)*)

*^2^ University of Antwerp, Electron Microscopy for Materials Science (EMAT), Department of Physics, Groenenborgerlaan 171, B-2020 Antwerpen, Belgium;
Dr. V. Samaee ([vahid.samaee@uantwerpen.be](mailto:vahid.samaee@uantwerpen.be)),
Prof. dr. D. Schryvers (*[*nick.schryvers@uantwerpen.be*](mailto:nick.schryvers@uantwerpen.be)*)*

^3^ *Academy of Sciences of the Czech Republic*, Institute of Thermomechanics, Dolejškova 5, 182000 Prague, Czech Republic;
Dr. J. Nejezchlebová ([*jitkanej@it.cas.cz*](mailto:jitkanej@it.cas.cz)),
Dr. P. Sedlák ([*petr.sedlak@fjfi.cvut.cz*](mailto:petr.sedlak@fjfi.cvut.cz))

^4^ Jozef Stefan Institute, Department for Nanostructures Materials,
*Jamova c. 39, SI-1000 Ljubljana, Slovenia;*Dr. A. Iveković ([*aljaz.ivekovic@ijs.si*](mailto:aljaz.ivekovic@ijs.si))

^5^ KU Leuven, PMA, Department of Mechanical Engineering,
Celestijnenlaan 300, B-3001 Leuven, Belgium;
Prof. dr. B. Van Hooreweder ([*brecht.vanhooreweder@kuleuven.be*](mailto:brecht.vanhooreweder@kuleuven.be))

*^6^* KU Leuven, Department of Materials Engineering,
Kasteelpark Arenberg 44, B-3001 Leuven, Belgium;
Prof. dr. K. Vanmeensel (*[kim.vanmeensel@kuleuven.be](mailto:kim.vanmeensel@kuleuven.be)*)

# Appendix

**Table A1** Transversal-isotropic elastic constants of LPBF-processed AlSi10Mg at room temperature (the symmetry axis x_3_ is aligned with the building direction). Average (Reuss) elastic properties: Young’s Modulus (E), Shear Modulus (G), Poisson ratio (ν), and elastic anisotropy factor (A), are calculated with these elastic constants.

|  | **Elastic constants** | | | | | | **Elastic properties** | | | |
| --- | --- | --- | --- | --- | --- | --- | --- | --- | --- | --- |
| **[GPa]** | **C_11_** | **C_33_** | **C_12_** | **C_13_** | **C_44_** | **C_66_^1^** | **E** | **G** | **ν [-]** | **A [-]** |
| **AB** | 102.6±0.5 | 102.7±0.6 | 52.4±0.7 | 52.9±0.7 | 25.8±0.3 | 25.1±0.6 | 67.7 | 25.3 | 0.337 | 1.04 |
| **DA** | 107.0±0.5 | 107.6±0.7 | 54.5±0.6 | 56.1±0.9 | 26.7±0.2 | 26.3±0.6 | 70.3 | 26.3 | 0.339 | 1.04 |
| **SR** | 108.9±0.5 | 108.9±0.7 | 56.9±0.6 | 56.1±0.9 | 27.1±0.2 | 26.0±0.6 | 71.1 | 26.5 | 0.340 | 1.04 |

^1^ C_66_ calculated as C_66_ = (C_11_ – C_12_)/2


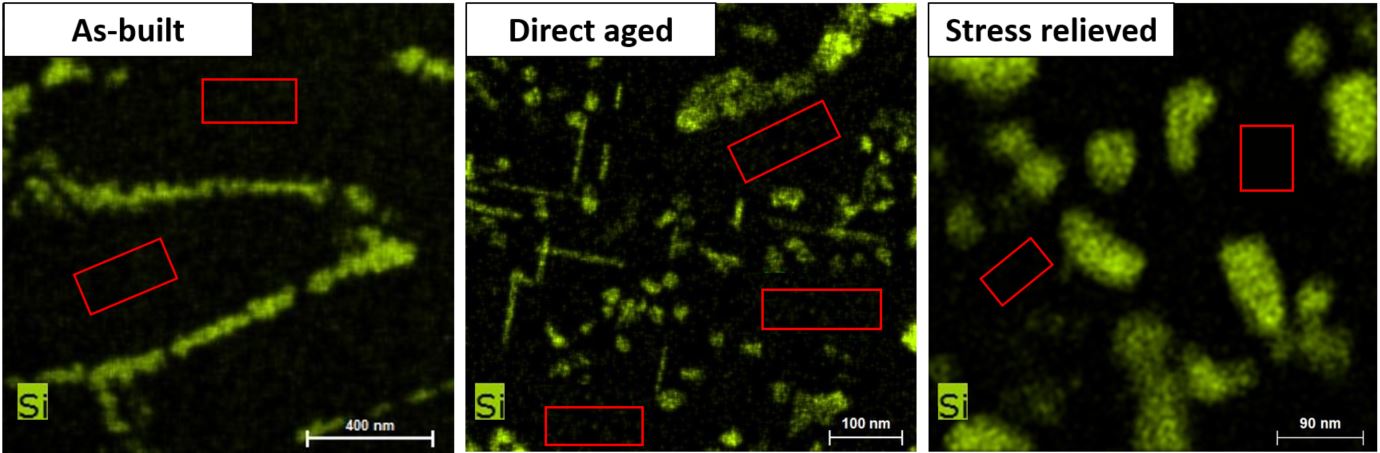


**Fig. A1** STEM-EDX images of the conditions (AB, DA, SR) showing the selection of areas used for the local elemental chemical quantification (Al, Si, Mg).

**Table A2** Brody-Flemings microsegregation model predicting the regime of the solidification front and solid concentration profile which is determined by the microsegregation parameter, a, and partition coefficient, k [18].

| **a** | **k** | **Solid concentration profile** $\boldsymbol{C}_{\boldsymbol{s}}$ | **Solidification front** |
| --- | --- | --- | --- |
| $\left\vert\boldsymbol{a} \right\vert\boldsymbol{\cong}\left\vert\frac{\boldsymbol{k}_{\boldsymbol{0}}\boldsymbol{-1}}{\boldsymbol{k}_{\boldsymbol{0}}} \right\vert$ | $\boldsymbol{k=}\boldsymbol{k}_{\boldsymbol{0}}$ | $\boldsymbol{C}_{\boldsymbol{s}}\boldsymbol{=}{\boldsymbol{k}_{\boldsymbol{0}}\boldsymbol{C}_{\boldsymbol{0}}\left[ \frac{\boldsymbol{a}}{\boldsymbol{k}_{\boldsymbol{0}}\boldsymbol{-1}} \right]\boldsymbol{\cong C}}_{\boldsymbol{0}}$ | Planar |
| $\left\vert\boldsymbol{a} \right\vert\boldsymbol{<}\left\vert\frac{\boldsymbol{k}_{\boldsymbol{0}}\boldsymbol{-1}}{\boldsymbol{k}_{\boldsymbol{0}}} \right\vert$ | $\boldsymbol{k}_{\boldsymbol{0}}\boldsymbol{<k<1}$ | $\boldsymbol{C}_{\boldsymbol{s}}\boldsymbol{=}\boldsymbol{k}_{\boldsymbol{0}}\boldsymbol{C}_{\boldsymbol{0}}\left[ \frac{\boldsymbol{a}}{\boldsymbol{k}_{\boldsymbol{0}}\boldsymbol{-1}}\boldsymbol{+}\left( \boldsymbol{1-}\frac{\boldsymbol{a}\boldsymbol{k}_{\boldsymbol{0}}}{\boldsymbol{k}_{\boldsymbol{0}}\boldsymbol{-1}} \right)\left( \boldsymbol{1-}\boldsymbol{f}_{\boldsymbol{s}} \right)^{\boldsymbol{k}_{\boldsymbol{0}}\boldsymbol{-1}} \right]$ | Cellular |
| $\left\vert\boldsymbol{a} \right\vert\boldsymbol{<}\left\vert\frac{\boldsymbol{k}_{\boldsymbol{0}}\boldsymbol{-1}}{\boldsymbol{k}_{\boldsymbol{0}}} \right\vert\boldsymbol{\ll1}$ | $\boldsymbol{k}_{\boldsymbol{0}}\boldsymbol{<k<1}$ | $\boldsymbol{C}_{\boldsymbol{s}}\boldsymbol{=}\boldsymbol{k}_{\boldsymbol{0}}\boldsymbol{C}_{\boldsymbol{0}}\left[ \frac{\boldsymbol{a}}{\boldsymbol{k}_{\boldsymbol{0}}\boldsymbol{-1}}\boldsymbol{+}\left( \boldsymbol{1-}\frac{\boldsymbol{a}\boldsymbol{k}_{\boldsymbol{0}}}{\boldsymbol{k}_{\boldsymbol{0}}\boldsymbol{-1}} \right)\left( \boldsymbol{1-}\boldsymbol{f}_{\boldsymbol{s}} \right)^{\boldsymbol{k}_{\boldsymbol{0}}\boldsymbol{-1}} \right]$ | Columnar dendritic |


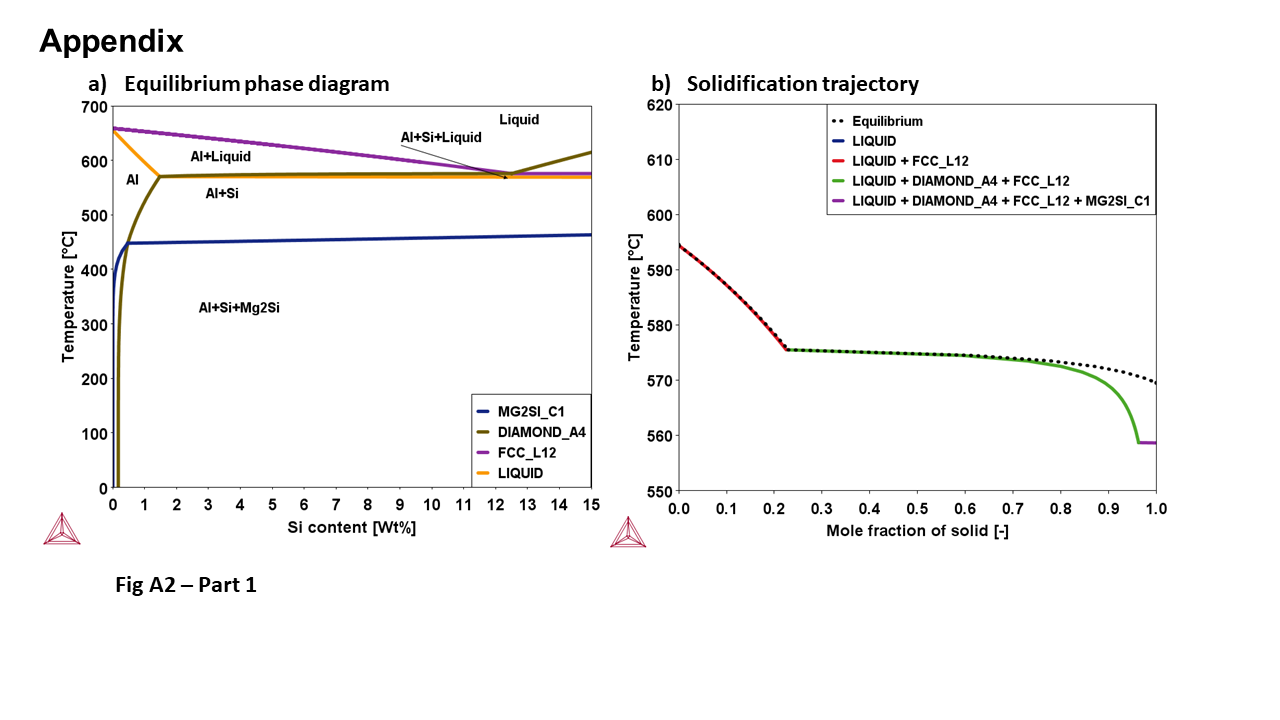

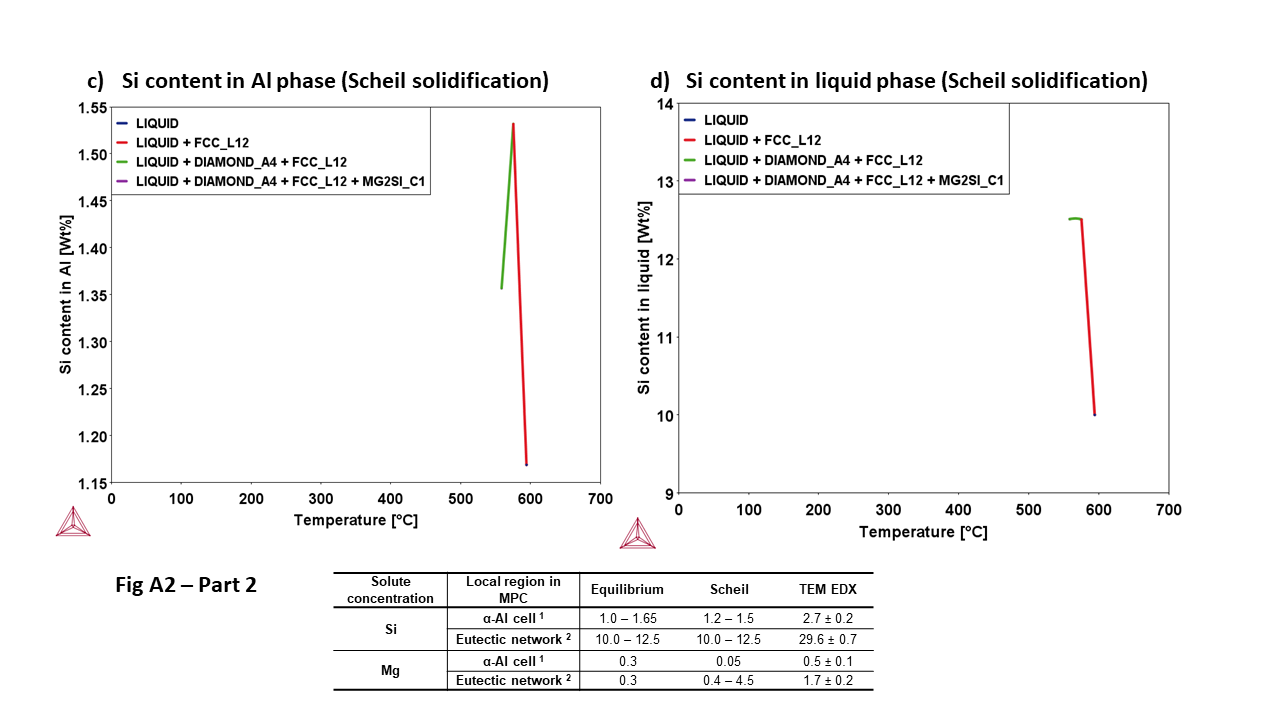

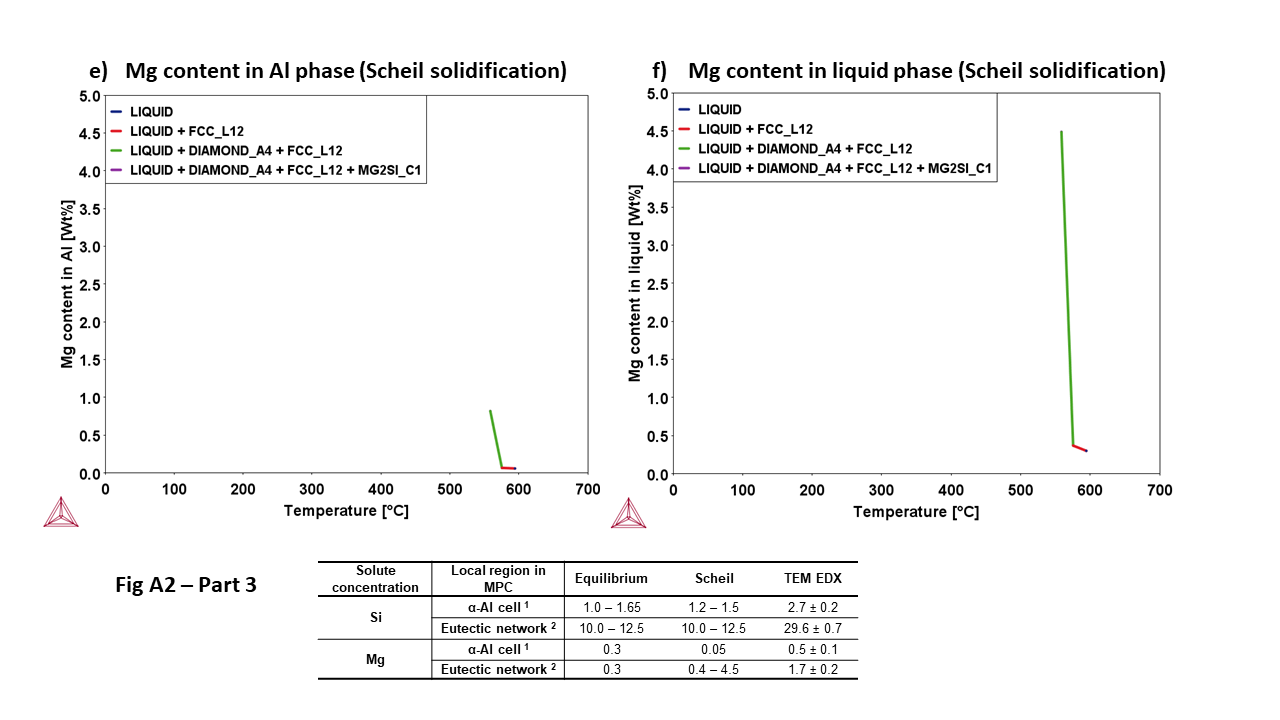


**Fig. A2** Equilibrium and Scheil solidification of AlSi10Mg0.3, predicted by Thermo-Calc 2020b software using the TCAL5.1 database. a) Equilibrium phase diagram, b) solidification trajectory under equilibrium and Scheil solidification conditions, (c-f) Si and Mg solute concentration profiles in respective primary Al (c, e) and in the last fraction of liquid phase (d, f), simulated under Scheil solidification conditions.
